# Added Value of Abnormal Lymph Nodes Detected with FDG-PET/CT in Suspected Vascular Graft Infection

**DOI:** 10.3390/biology12020251

**Published:** 2023-02-05

**Authors:** Nick D. van Rijsewijk, Jasper H. G. Helthuis, Andor W. J. M. Glaudemans, Marjan Wouthuyzen-Bakker, Niek H. J. Prakken, David J. Liesker, Ben R. Saleem, Riemer H. J. A. Slart

**Affiliations:** 1 Medical Imaging Center, Department of Nuclear Medicine and Molecular Imaging, University Medical Center Groningen, University of Groningen, 9713 GZ Groningen, The Netherlands; 2 Medical School Twente, Medisch Spectrum Twente, 7512 KZ Enschede, The Netherlands; 3 Department of Medical Microbiology and Infection Prevention, University Medical Center Groningen, University of Groningen, 9713 GZ Groningen, The Netherlands; 4 Medical Imaging Center, Department of Radiology, University Medical Center Groningen, University of Groningen, 9713 GZ Groningen, The Netherlands; 5 Department of Vascular Surgery, University Medical Center Groningen, University of Groningen, 9713 GZ Groningen, The Netherlands; 6 Faculty of Science and Technology, Department of Biomedical Photonic Imaging, University of Twente, 7522 NB Enschede, The Netherlands

**Keywords:** vascular graft/endograft infection, lymph nodes, ^18^F-FDG PET/CT

## Abstract

**Simple Summary:**

Patients with a suspected vascular graft and endograft infection regularly undergo a medical imaging examination with radioactive labelled glucose as a tracer (^18^F-fluorodeoxyglucose positron emission tomography/computed tomography) for diagnosis. Nuclear medicine physicians currently use the intensity and uptake pattern of the tracer around the prosthesis as an indication for infection. This study aimed to investigate the added value of possibly present lymph nodes in the diagnosis of vascular graft infection. Uptake and enlargement of lymph nodes is highly indicative for vascular graft infections but does not add to existing interpretation criteria.

**Abstract:**

Vascular graft and endograft infections (VGEI) cause a serious morbidity and mortality burden. ^18^F-fluorodeoxyglucose positron emission tomography/computed tomography (^18^F-FDG PET/CT) imaging is frequently used in the diagnostic workup, but the additional value of abnormal (^18^F-FDG active and/or enlarged) locoregional lymph nodes is unknown. In this retrospective study, the additional diagnostic value of abnormal locoregional lymph nodes on ^18^F-FDG PET/CT imaging for VGEI was evaluated, including 54 patients with a culture-proven VGEI (defined according to the Management of Aortic Graft Infection [MAGIC] group classification) and 25 patients without VGEI. ^18^F-FDG PET/CT was qualitatively and quantitatively assessed for tracer uptake and pattern at the location of the vascular graft, and locoregional lymph node uptake and enlargement (>10 mm). ^18^F-FDG uptake intensity and pattern independently predicted the presence of VGEI by logistic regression (Χ^2^: 46.19, *p* < 0.001), with an OR of 7.38 (95% CI [1.65, 32.92], *p* = 0.009) and 18.32 (95% CI [3.95, 84.88], *p* < 0.001), respectively. Single visual assessment of abnormal locoregional lymph nodes predicted the presence of VGEI with a sensitivity of 35%, specificity of 96%, PPV of 95%, and NPV of 41%. The visual assessment of abnormal lymph nodes after qualitative assessment of ^18^F-FDG uptake intensity and pattern at the vascular graft location did not independently predict the presence of VGEI by logistic regression (Χ^2^: 3.60, *p* = 0.058; OR: 8.25, 95% CI [0.74, 63.37], *p* = 0.096). In conclusion, detection of abnormal locoregional lymph nodes on ^18^F-FDG PET/CT has a high specificity (96%) and PPV (95%) for VGEI. However, it did not add to currently used ^18^F-FDG PET/CT interpretation criteria.

## 1. Introduction

Although there is a tendency from open surgery to more endovascular procedures, vascular graft and endograft infection (VGEI) still occurs in approximately up to 6% of all vascular graft replacements [1,2]. Early postoperative infections mainly occur due to intraoperative contamination or a direct progression of a wound infection to the graft, while VGEI occurring a long time after surgery is considered to be a result of the reactivation of a previously undetected infection of the graft or seeding of the graft due to bacteraemia [3]. As a complication, VGEI results in serious morbidity and mortality. For instance, limb loss is noted up to 27% in extra-anatomic reconstructions for abdominal aortic VGEI cases and mortality rates up to 75% are observed in patients with a thoracic aortic VGEI [4].

Early diagnosis and treatment using targeted antibiotics, percutaneous drainage, or surgical intervention are essential. However, particularly at an early stage, VGEI diagnosis is difficult. Clinical signs such as fever, pain, erythema, pulsatile groin mass, and laboratory blood tests for infection parameters (white blood cell count, C-reactive protein (CRP), erythrocyte sedimentation rate (ESR)) have low predictive value, and later stages commonly present with bleeding, sepsis, or haemorrhagic shock [2,4].

Therefore, early evaluation using diagnostic imaging techniques is important when clinical suspicion of VGEI arises. In the most recent guideline from the European Society of Vascular Surgery, the first-choice imaging modality is computed tomography angiography (CTA), or magnetic resonance angiography (MRA) when CTA is contra-indicated. ^18^F-fluorodeoxyglucose positron emission tomography/computed tomography (^18^F-FDG PET/CT) is recommended when VGEI is still suspected after a negative or inconclusive CTA [4]. Furthermore, in a recently published structural clinical approach for the diagnosis and treatment of VGEI, ^18^F-FDG PET/CT imaging is recommended for all suspected VGEIs when the interval between the index surgery is more than three months; CTA is recommended in early postoperative suspected VGEI [5]. Other imaging modalities to consider in the diagnostic workup of VGEI are ultrasonography (US) and radiolabelled white blood cell (WBC) scintigraphy [6].

Although ^18^F-FDG PET/CT is recommended in some of the structured literature as a diagnostic method for VGEI [4,5], a standardized interpretation and reporting criteria for VGEI on ^18^F-FDG PET/CT is lacking. Existing interpretation criteria are based on tracer uptake patterns (homogeneous or heterogeneous), a visual grading scale (VGS), and sometimes semi-quantitative analysis by calculating the standardized uptake value (SUV) or target-to-background ratio (TBR) [1]. 

It is known that lymph nodes can be enlarged in several infectious diseases, showing increased ^18^F-FDG uptake [7,8,9,10]. However, assessment of ^18^F-FDG uptake in locoregional lymph nodes is not commonly included as an interpretation criterion for ^18^F-FDG PET/CT imaging in VGEI [4]. Evaluation of ^18^F-FDG uptake and size of locoregional lymph nodes in addition to the current interpretation criteria for VGEI might increase its diagnostic accuracy. 

This study therefore aimed to evaluate the (additional) diagnostic value of abnormal locoregional lymph nodes (with an increased ^18^F-FDG uptake and/or an increased diameter) on ^18^F-FDG PET/CT imaging of patients with suspected VGEI.

## 2. Materials and Methods

### 2.1. Study Population

In this retrospective single-center study, the electronic patient record (EPR) system was consulted to include adult patients with suspected abdominal and/or inguinal VGEI referred to the nuclear medicine department for ^18^F-FDG PET/CT imaging. In addition, patients referred for ^18^F-FDG PET/CT imaging for other reasons with a documented vascular graft or endograft in situ (without a suspected infection), were included as the other referral group. Definite VGEI diagnosis was defined according to the Management of Aortic Graft Infection [MAGIC] group classification with at least one surgical or laboratory major criterion and one minor criterion from another category [11], and served as the primary outcome measurement. Patients with suspected VGEI infection were excluded if data on microbiological culture and/or surgical findings matching for prosthesis infection were missing. The local Medical Ethical Commission approved this retrospective study without the need for informed consent (METC number 202000143).

For all included patients, data on gender, age, body mass index (BMI), injected tracer activity, blood glucose levels before tracer administration, possible confounders (smoking, diabetes, hypertension, hyperlipemia, cardiac status, pulmonary status, and renal status), clinical symptoms, microbiology sampling results, antibiotic usage, surgical procedurals (dichotomous; open or endovascular procedure), and graft location (aortoiliac, iliofemoral, other) were acquired. Based on microbiological culture and/or surgical findings, a diagnosis of definite VGEI was made. Classifications of possible confounders were applied according to the common reporting standard of the Society of Vascular Surgery [12].

### 2.2. PET/CT Acquisition and Image Analysis

^18^F-FDG PET/CT scans were acquired from vertex to mid-thigh using three different PET/CT scanners (Biograph Vision, mCT40 and mCT64, Siemens Healthineers, Erlangen, Germany). Patients received a standard intravenous administration of 3 MBq per kilogram dosage of ^18^F-FDG according to EANM guidelines [7]. Fasting with access to non-caloric beverages was accomplished in all patients for at least 6 h prior to scanning. PET images were acquired with 3 min per bed position. Scans were performed with a time-interval of 60 ± 5 min after injection of ^18^F-FDG. An additional continuous breathing low dose CT (80–120 kV, 20–35 mAs, and 5 mm slice thickness) was performed for attenuation correction and visualization of anatomical structures.

^18^F-FDG PET/CT images were assessed both qualitatively and semi-quantitatively. The qualitative assessment was based on a VGS and was performed both at the location of the graft and at the level of the locoregional lymph nodes corresponding with suspected lymphatic drainage pathways [13,14,15]. The visual 4-point grading scale classified the ^18^F-FDG uptake: grade 1—no ^18^F-FDG uptake; grade 2—mild ^18^F-FDG uptake, comparable to inactive musculature and fat; grade 3—moderate ^18^F-FDG uptake, clearly above inactive musculature and fat; and grade 4—high ^18^F-FDG uptake, comparable with physiological ^18^F-FDG activity in the bladder [16]. A grade 3 or 4 ^18^F-FDG uptake at the graft location was considered positive for infection on ^18^F-FDG PET/CT. Additionally, the ^18^F-FDG uptake pattern (homogenous/diffuse or heterogenous/focal) was noted for the site of the vascular graft. Locoregional lymph nodes in the corresponding suspected lymphatic drainage pathway were identified and had to match at least one of the two following criteria to be defined as abnormal: 1. a visual ^18^F-FDG uptake of grade 3 or 4, and/or 2. a short axis diameter larger than 10 mm on low-dose CT. 

Semi-quantitative analysis was performed on reconstructed images following standardized criteria: NEDPAS (scans < 2015) or EARL1 (scans > 2015) criteria [17,18]. Hence, spherical and cylindrical volumes of interests were used to measure the SUV, which were corrected for blood glucose. SUV_max_ and SUV_peak_ were measured at the site of the vascular graft and at the abnormal lymph nodes. SUV_mean_ measurements were performed in the liver, spleen, and bone marrow (lumbar spine, region L5). 

All measurements were performed by a nuclear medicine physician in training (JH) using Syngo.via VB30 software (Siemens Healthineers, Erlangen, Germany), except for ^18^F-FDG uptake patterns at the site of the vascular graft, which were jointly determined by two experienced nuclear medicine physicians (RS, AG). Uncertainties were discussed and solved by consensus with an experienced nuclear medicine physician (RS) and an additional sample check was performed by a physician researcher (NvR). 

### 2.3. Statistical Analysis

Continuous variables for the whole study population were assumed to be normally distributed according to the central limit theorem and presented as mean with standard deviation. For frequencies, absolute numbers with percentages were used. Descriptive statistics were performed to provide an overview of patients’ characteristics within the three subgroups: patients with suspected VGEI and proven infection based on intraoperative findings, patients with a suspected VGEI without infection based on intraoperative findings, and a group of patients with other reasons for referral. Comparison between groups was performed using one-way ANOVAs for continuous variables, Fisher-Freeman-Halton exact tests for binary variables, and Chi-square tests for non-binary categorical variables. 

For further analysis, two subgroups were created: patients with a proven VGEI and patients without a VGEI. The group of patients without VGEI consisted of the patients with suspected VGEI, but in whom infection was ruled out based on intraoperative findings, and the group of patients with other reasons for referral. Comparison of the qualitative evaluation of the vascular graft and the lymph nodes was performed between the two groups using two-tailed Fisher’s Exact test. Logistic regression was used to evaluate the predictive value of qualitative assessment of ^18^F-FDG uptake and pattern at the site of vascular graft and the (additional) value of abnormal lymph nodes in VGEI. Sensitivity, specificity, positive predictive value (PPV), and negative predictive value (NPV) were calculated for the qualitative assessment of the abnormal lymph nodes: the presence of abnormal lymph nodes were classified as true positive (TP) if accompanied with microbiological evidence for VGEI, while microbiological evidence for a VGEI without abnormal lymph nodes was considered to be false negative (FN). Cases without VGEI showing abnormal lymph nodes were defined as false positive (FP) results. All other referral cases without abnormal lymph nodes were considered to be true negative (TN), as well as the cases with a suspected infection without microbiological evidence for VGEI showing no abnormal lymph nodes.

For quantitative assessment an independent-samples t-test was performed to identify significant differences between patients with and without a VGEI. 

All results were considered statistically significant with a *p*-value <0.05. Missing data were excluded pairwise. Statistical analyses were performed using IBM^®^ SPSS^®^ Statistics (Version 28, IBM Corp., Armonk, NY, USA).

## 3. Results

### 3.1. General

A potential number of 116 patients were identified, of which 37 were excluded for several reasons: the final diagnosis of 30 patients was based on imaging and/or clinical features without microbiological evidence, five patients underwent a PET/CT examination before the vascular replacement surgery, and the final diagnosis was uncertain in two patients. Finally, 79 patients were included in this retrospective study: 59 patients in the suspected VGEI group and 20 patients in the other referral group. Within the suspected VGEI group, 54 patients had a final diagnosis of VGEI, and 5 patients had no infection. Characteristics of the study population are summarized in Table 1. There were no significant differences between the subgroups, except for hypertension and hyperlipidemia in medical history, type of surgical procedure, and use of antibiotics before the PET/CT examination.

### 3.2. Qualitative Assessment

Visual assessment of ^18^F-FDG uptake at the site of the vascular graft was marked positive in 57 patients (72.2%) and was significantly more frequent in patients with VGEI (90.7% versus 32.0%). The uptake pattern was also significantly different in patients with or without a VGEI. Heterogeneous uptake at the site of the vascular graft was seen in a total of 47 patients (59.5%), of which 44 patients were diagnosed with a VGEI (93.6%). 

Abnormal lymph nodes were detected in a total of 20 patients with ^18^F-FDG PET/CT imaging: 11 cases met the criteria to be defined as positive for ^18^F-FDG uptake and 16 cases met the criteria for enlargement, seven patients showed both increased ^18^F-FDG uptake and enlarged lymph nodes. None of the patients without a VGEI had lymph nodes positive for ^18^F-FDG uptake. The criteria for enlargement were met in a single patient without a VGEI, and this patient belonged to the other referral subgroup. Visual assessment of ^18^F-FDG uptake and enlargement of lymph nodes, as single entities and combined, were significantly different between the infection and non-infection group. An overview is given in Table 2.

Qualitative assessment of the ^18^F-FDG uptake intensity and pattern at the location of the vascular graft independently predicted the presence of VGEI by logistic regression (Χ^2^: 46.19, *p* < 0.001), with an odds ratio (OR) of 7.38 for ^18^F-FDG uptake intensity (95% CI [1.65, 32.92], *p* = 0.009) and an OR of 18.32 for uptake pattern (95% CI [3.95, 84.88], *p* < 0.001). 

In the single visual assessment, 19 cases were classified as TP, 24 cases as TN, 35 cases as FN, and 1 case as FP, leading to a sensitivity, specificity, PPV, and NPV of 35.2%, 96.0%, 95.0%, and 40.7%, respectively. This assessment of abnormal lymph nodes predicted the presence of VGEI by logistic regression (Χ^2^: 10.95, *p* < 0.001; OR: 13.03, 95% CI [1.63, 103.96], *p* = 0.015). However, this visual assessment of abnormal lymph nodes after the qualitative assessment of ^18^F-FDG uptake intensity and pattern at the location of the vascular graft did not independently predict the presence of VGEI by logistic regression (Χ^2^: 3.60, *p* = 0.058; OR: 8.25, 95% CI [0.74, 63.37], *p* = 0.096).

### 3.3. Semi-Quantitative Assessment

**Figure 1:**
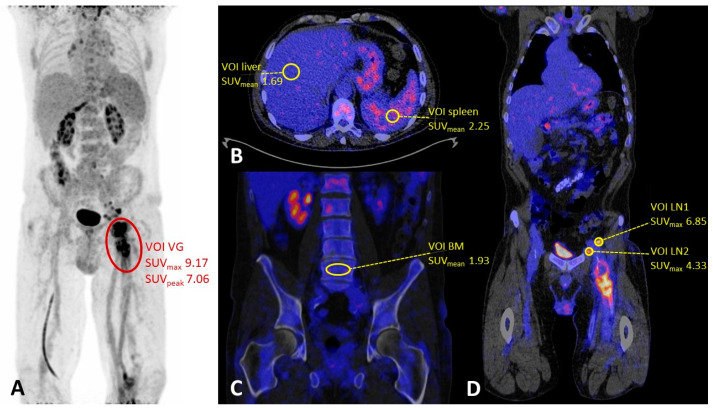
(**A**) Maximum intensity projection (MIP) of a patient with a proven femoral vascular graft infection (grade 3, heterogeneous uptake) with multiple lymph nodes on the suspected external and common iliac lymphatic drainage pathway. (**B**) SUV_mean_ measurements in the liver and the spleen. (**C**) Bone marrow SUV_mean_ measurement in the lumbar spine (L5). (**D**) SUV_max_ measurements in the lymph nodes. Abbreviations: VOI = volume of interest, VG = vascular graft, BM = bone marrow, LN = lymph node.

Five scans had to be excluded for semi-quantitative analysis, no NEDPAS or EARL reconstructions were available for four patients, and for one patient, the blood glucose level at the time of ^18^F-FDG injection was unknown. SUV_max_ and SUV_peak_ were significantly higher in patients with an ongoing infection at the site of the vascular graft. The SUV_max_ of the lymph nodes was not significantly different between the infection and non-infection groups, but there was only one scan showing lymph nodes in the group without infection. SUV_mean_ in the liver, the spleen, and the bone marrow were also not significantly different between the two groups. A summary of the quantitative PET/CT imaging characteristics is shown in Table 3 and an example of the measurements is given in Figure 1.

## 4. Discussion

Strict interpretation and reporting criteria for diagnosing a VGEI at ^18^F-FDG PET/CT are lacking [4,7]. Visual grading of intensity and uptake pattern of ^18^F-FDG around the vascular prosthesis are often used as an indicator of VGEI [1]. Abnormal locoregional lymph nodes detected at ^18^F-FDG PET/CT might help to further improve diagnostic accuracy. To the best of our knowledge, this is the first study defining the additional diagnostic value of abnormal locoregional lymph nodes at ^18^F-FDG PET/CT for diagnosing VGEI.

Diagnostic accuracy of qualitative assessment of the vascular graft in ^18^F-FDG PET/CT imaging for suspected VGEI has been investigated in multiple meta-analyses. Using a visual grading scale for ^18^F-FDG uptake at the site of the vascular graft, a pooled sensitivity of 89% and 90% was observed, together with a pooled specificity of 61% and 59%. For the uptake pattern, pooled sensitivity was 89% and 94%, and pooled specificity was 78% and 81% [1,19]. As expected, the uptake pattern was significantly different between the group with and without a VGEI in our study, with comparable sensitivities and specificities for uptake intensity (91% and 68%, respectively) and uptake pattern (81% and 88%, respectively). Both independently predicted the presence of VGEI with an OR of 7.38 for ^18^F-FDG uptake intensity and an OR of 18.32 for uptake pattern.

We observed that single visual assessment of abnormal lymph nodes predicts the presence of VGEI by logistic regression with an odds ratio of 8.25 and a high specificity (96%) and PPV (95%). Only one case in the non-infection group (other referral subgroup) met the criteria for enlargement and no cases without VGEI had a grade 3 or 4 tracer uptake. Despite the high odds ratio and high specificity for VGEI of abnormal lymph nodes as a singular entity, there was no added value of them to the current diagnostic ^18^F-FDG PET/CT criteria. Possible explanations could be that most patients with abnormal lymph nodes also show increased ^18^F-FDG uptake in the vascular graft with a heterogeneous appearance, thus it did not provide extra information on top of the already existing interpretation criteria, or that the sample size is not sufficient in our study. 

Despite the high specificity, the sensitivity of detecting abnormal locoregional lymph nodes is low. Patients with a VGEI do not necessarily demonstrate abnormal lymph nodes on ^18^F-FDG PET/CT, resulting in a low sensitivity. Another possible cause of low sensitivity of abnormal lymph nodes could be the result of using antibiotics before ^18^F-FDG PET/CT examination [20], as patients with a suspected VGEI are rapidly treated with antibiotics.

Specificity of abnormal lymph nodes can be influenced by, for example, inflammatory diseases and malignancies [20,21], which can lead to false positive results for the diagnosis of VGEI. As ^18^F-FDG is an analog of glucose, it is a non-specific tracer which is taken up via the cell membrane glucose transporter by all kinds of living cells [7,20]. Therefore, initially, specificity of lymph nodes was not estimated to be high. However, only one of 20 patients with abnormal lymph nodes on ^18^F-FDG PET/CT examination classified as false positive for VGEI. As this patient did have a medical history of multiple abdominal surgical interventions including a urostomy, this lymph node was highly suspected to be reactive due to other ongoing inflammatory processes. Alternative causes for lymph node activity should always be taken into account.

A systemic review by Reinders-Folmer et al. suggested that the diagnostic performance of WBC scintigraphy combined with SPECT/CT was the best in VGEI patients [1]. However, due to possible availability issues and technique-specific characteristics, such as the time-consuming process of WBC labelling, prolonged acquisition time, the requirement of highly trained personnel, and possible exposure to infected blood products, ^18^F-FDG PET/CT is a good and adequate alternative [1]. Nonetheless, in light of our study, WBC scintigraphy may be able to distinguish reactive inflammatory lymph nodes and abnormal lymph nodes due to infection of the vascular graft.

One of the major limitations of this study is its retrospective study design: all patients referred for ^18^F-FDG PET/CT in our university medical center were highly suspected for infection, as only 5 out of 59 referred patients did not have an actual VGEI. Furthermore, to obtain a large enough sample size for statistical analysis, there was a need to add a group of patients with a vascular graft or endograft in situ who were referred for ^18^F-FDG PET/CT for other reasons than a suspected VGEI. This may have resulted in a selection bias, especially within the non-infected group, as clinical symptoms suggestive for VGEI may also be caused by other pathological processes in proximity to the vascular graft. Nonetheless, the other referral group and patients with a suspected VGEI were relatively comparable in regard to patient characteristics, which likely still warrant using them as negative cases to aid in statistical analysis.

Further research is needed to evaluate the diagnostic accuracy of lymph nodes for detecting and diagnosing VGEI. A prospective study using a gold standard would be particularly useful to provide more evidence and valuable insight into the diagnostic accuracy of abnormal lymph nodes. As technology continues to advance, improved camera systems (such as long axial field of view PET systems) and the development of new tracers may help to improve the diagnostic accuracy [22,23]. The application of a diagnostic CT instead of a low dose CT may also improve optimal detection and measurements of lymph nodes. Additionally, the development of more sensitive methods may be necessary to accurately diagnose VGEI, since abnormal lymph nodes are especially present in fulminant infections. Finally, PET reporting and the interpretation in VGEI should be more standardized and harmonized, taking the lymph node status into account as well. 

## 5. Conclusions

The presence of abnormal locoregional lymph nodes at ^18^F-FDG PET/CT, defined as moderate to high tracer uptake and/or size of lymph nodes >10 mm on low-dose CT, has a high specificity (96%) and PPV (95%) for vascular graft or endograft infection. However, we found no additional value of the detection of abnormal lymph nodes to the current ^18^F-FDG PET/CT interpretation criteria for vascular graft infections.

## Figures and Tables

**Table 1:** Main characteristics of the study population. A comparison has been made between the three subgroups for each characteristic and significant differences are indicated in bold. * A total of 7 missing values: 3 missing values in the other referral group and 4 missing values in the proven infection subgroup. ** One missing value.

		Suspected Infection (*n* = 59)				
		Proven Infection	Without Infection	Other Referral	Total	*p*
**General characteristics**						
Number of patients (%)		54 (68.4)	5 (6.6)	20 (25.0)	79 (100)	
Age—mean (SD) [years]		68.6 (8.7)	73.6 (7.4)	67.9 (7.4)	68.7 (8.4)	0.383
Gender—*n* male (%)		43 (79.6)	5 (100)	17 (85.0)	65 (82.3)	0.694
Body mass index—mean (SD)		26.3 (4.3)	24.5 (2.7)	25.2 (3.9)	25.9 (4.1)	0.448
**Medical history**						
Tobacco usage—*n* (%) *						0.858
	*Non-smoker*	22 (44.0)	1 (20.0)	7 (41.2)	30 (41.7)	
	*Former smoker*	4 (8.0)	1 (20.0)	2 (11.8)	7 (9.7)	
	*Current smoker*	24 (48.0)	3 (60.0)	8 (47.1)	35 (48.6)	
Diabetes—*n* (%)		18 (33.3)	1 (20.0)	2 (10.0)	21 (26.6)	0.106
Hypertension—*n* (%)		25 (46.3)	3 (60.0)	16 (80.0)	44 (55.7)	0.027
Hyperlipidemia—*n* (%)		13 (24.1)	4 (80.0)	12 (60.0)	29 (36.7)	0.002
Cardiac status—*n* (%)		32 (59.3)	4 (80.0)	10 (50.0)	46 (58.2)	0.507
Pulmonary status—*n* (%)		16 (29.6)	2 (40.0)	6 (30.0)	24 (30.4)	0.920
Renal status—*n* (%)		15 (27.8)	0 (0)	9 (45.0)	24 (30.4)	0.118
**Surgical characteristics**						
Type of surgical procedure—*n* (%)						0.011
	*Open*	43 (79.6)	3 (60.0)	9 (45.0)	55 (69.6)	
	*Endovascular*	11 (20.4)	2 (40.0)	11 (55.0)	24 (30.4)	
Graft location—*n* (%)				**		0.315
	*Aortoiliac*	18 (33.3)	3 (60.0)	11 (57.9)	32 (40.5)	
	*Iliofemoral*	19 (35.2)	1 (20.0)	3 (15.8)	23 (29.1)	
	*Other*	17 (31.5)	1 (20.0)	5 (26.3)	23 (29.1)	
Time from last surgery to PET imaging in months – mean (SD)		47.2 (54.0)	43.0 (31.4)	55.2 (50.9)	48.9 (51.8)	0.814
**Other clinical characteristics**						
Use of antibiotics before PET/CT scan—*n* (%)		44 (83.0) **	3 (75.0) **	5 (26.3) **	52 (68.4)	<0.001
Blood glucose level at time of ^18^F-FDG injection – mean (SD) [mmol/L]		5.7 (1.0) **	5.5 (0.9)	6.2 (1.9)	5.8 (1.3)	0.298

**Table 2:** Qualitative PET/CT imaging characteristics in vascular graft infection.

			Vascular Graft Infection			
			Yes (*n* = 54)	No (*n* = 25)	Total (*n* = 79)	*p*
**Vascular graft**						
	^18^F-FDG uptake—*n* (%)					< 0.001
		*Positive*	49 (90.7)	8 (32.0)	57 (72.2)	
		*Negative*	5 (9.3)	17 (68.0)	22 (27.8)	
	Uptake pattern—*n* (%)					< 0.001
		*Heterogeneous*	44 (81.5)	3 (12.0)	47 (59.5)	
		*Homogeneous*	10 (18.5)	22 (88.0)	32 (40.5)	
**Lymph nodes**						
	^18^F-FDG uptake—*n* (%)					0.014
		*Positive*	11 (20.4)	0 (0)	11 (13.9)	
		*Negative*	43 (79.6)	25 (100)	68 (86.1)	
	Enlarged (>10 mm)—*n* (%)					0.016
		*Yes*	15 (27.8)	1 (4.0)	16 (20.3)	
		*No*	39 (72.2)	24 (96.0)	63 (79.7)	
	Combined FDG uptake and enlarged—*n* (%)					0.002
		*Yes*	19 (35.2)	1 (4.0)	20 (25.3)	
		*No*	35 (64.8)	24 (96.0)	59 (74.7)	

**Table 3:** Quantitative PET/CT imaging characteristics in vascular graft infections (*n* = 74), expressed in SUV values corrected for glucose. NEDPAS or EARL reconstructions were unavailable for four patients, and one patient had missing data on blood glucose level, and these were therefore excluded. * Single patient.

		Vascular Graft Infection		*p*
		Yes (*n* = 49)	No (*n* = 25)	
**Vascular graft—mean (SD)**				
	SUV_max_	9.48 (4.03)	5.37 (2.50)	<0.001
	SUV_peak_	7.27 (3.24)	4.37 (2.05)	<0.001
**Lymph nodes (*n* = 20)—mean (SD)**				
	SUV_max_	4.16 (1.61)	2.46 *	0.321
**SUV_mean_—mean (SD)**				
	Liver	2.50 (0.64)	2.73 (1.01)	0.318
	Spleen	2.47 (0.58)	2.40 (0.84)	0.710
	Bone marrow	2.51 (0.90)	2.11 (0.81)	0.062

## Data Availability

The data presented in this study are available on request from the corresponding author exclusively for the purpose of reproducing the study results. The data are not publicly available due to privacy reasons.
